# Social isolation and its impact on child and adolescent development: a systematic review

**DOI:** 10.1590/1984-0462/2022/40/2020385

**Published:** 2021-10-04

**Authors:** Isabelle Lina de Laia Almeida, Jaqueline Ferraz Rego, Amanda Carvalho Girardi Teixeira, Marília Rodrigues Moreira

**Affiliations:** a Universidade Federal de Uberlândia, Uberlândia, Minas Gerais, Brazil.

**Keywords:** Social isolation, Quarantine, Child development, Adolescent development, COVID-19, Isolamento social, Quarentena, Desenvolvimento infantil, Desenvolvimento do adolescente, COVID-19

## Abstract

**Objective:**

This study aims to analyze the effects of social isolation on children's and teenagers’ development, with emphasis on the possible impacts over their physical and mental health.

**Data source:**

Review of the literature following the standards of PRISMA using the SciELO, LILACS and PubMed databases. The following key-words were used: “social isolation” and “child development”, “quarantine” and “adolescent development” according to the Medical Subject Headings (MESH) and their translation to the Portuguese. Studies in English, Portuguese and Spanish from inception were included.

**Data synthesis:**

519 studies were screened and 12 were included in the systematic review. Five of those focused the psychology and social issues, two of them the effects of pandemics on these issues; four studies reported on impacts on general health and two consequences over the hypothalamus- hypophysis - adrenal axis and the cognitive and social development.

**Conclusions:**

The review shows a strong association between social isolation and anxiety and depression in children and adolescents. Social isolation leads to higher levels of cortisol and worse cognitive development. Therefore, the mental and physical health of children and adolescents need a careful follow up by health professionals during and after the COVID-19 pandemic.

## RESUMO

**Objetivo:**

Analisar os efeitos do isolamento social para o desenvolvimento de crianças e adolescentes, considerando consequências em médio e longo prazos, e entender possíveis impactos sobre a saúde mental e física.

**Fontes de dados:**

Revisão sistemática da literatura seguindo os parâmetros da lista Preferred Reporting Items for Systematic Reviews and Meta-Analysis (PRISMA) nas bases de dados Scientific Electronic Library Online (SciELO), Literatura Latino-Americana e do Caribe em Ciências da Saúde (LILACS) e PubMed. Os descritores utilizados foram “social isolation” AND “child development”, “quarantine” AND “child development”, “social isolation” AND “adolescent development”, “quarantine” AND “adolescent development” de acordo com o Medical Subject Headings (MeSH) e seus equivalentes para a língua portuguesa, conforme os Descritores em Ciências da Saúde (DeCS). Incluíram-se artigos originais em inglês, português e espanhol, sem delimitação temporal.

**Síntese dos dados:**

Identificaram-se 519 referências, e, após critérios de inclusão e exclusão, 12 artigos foram analisados. Cinco abordaram a questão psicossocial (sendo dois sobre os efeitos das pandemias), quatro sobre os impactos na saúde em geral, dois sobre consequências no eixo hipotálamo-hipófise-adrenal e um sobre o desenvolvimento cognitivo e social.

**Conclusões:**

Os artigos revisados evidenciaram forte relação entre isolamento social e maior incidência de sentimentos como ansiedade e depressão na população de crianças e adolescentes. Além disso, identificaram-se aumento nos níveis de cortisol e piora no desenvolvimento cognitivo dessa faixa etária. Logo, o acompanhamento da saúde mental e física desses jovens por profissionais da saúde deve estar presente durante e após a pandemia.

## INTRODUCTION

Since the beginning of 2020, the world has faced a serious global crisis resulted from the pandemic due to the infection of the new coronavirus, namely severe acute respiratory syndrome coronavirus 2 (SARS-CoV-2), which causes coronavirus disease 2019 (COVID-19), detected in China in December 2019.^1^ Distancing and social isolation were adopted worldwide as a non-pharmacological measure in order to face the pandemic, aiming to control the spread of the disease and the contamination of the population. It is known that these are the possible measures at the moment, but one should not lose sight of the fact that social distancing may have negative impacts at different levels and contexts of development.^2^

Pandemics, like other disasters, have been part of human history for centuries. However, the response to pandemics is necessarily distinguished, as it requires separation, isolation, and quarantine. In addition to the potential protective effects of the community attributed to quarantine, the risks for quarantined individuals need to be identified.^3^ Studies show that this situation influences people's daily behavior, causing anxiety, fear, depression, and panic.^4^ ^,^ ^5^

It is increasingly recognized that individuals who experience social isolation are at increased risk of disease. Adverse psychosocial experiences, such as social isolation, can be particularly harmful to developing children and adolescents.^6^ Social distancing can aggravate or generate functional and behavioral difficulties in this age group. It is also noted that this stress scenario alters physical activity and sleep, essential for general development. There is ample evidence that these factors have a profound impact on brain plasticity and, therefore, on cognitive and emotional development.^7^

Thus, the physical and mental health of children and adolescents should be a point of attention, considering that they are part of a vulnerable population.^8^ The situation of uncertainty generated by COVID-19 can cause anger, depression, and anxiety, given the loss contact with other people, the distancing, and the illness or death of family and friends. As the changes caused by COVID-19 are sudden and cover many aspects of our daily lives, these adverse reactions tend to worsen, impairing human reflexive function.^9^ Pandemic planning, therefore, must consider the needs of this population, especially, and of their families, ensuring that they do not suffer trauma in the long term due to the experience of pandemic diseases or public health response strategies.^10^

Considering the above and the current context of the COVID-19 pandemic, the objective of this work was to conduct a systematic literature review to address the effects of social isolation on the development of children and adolescents, taking into account the consequences in the medium and long term, as well as to understand the possible results of these actions on the human organism.

## METHOD

This review article was prepared according to the items in the Preferred Reporting Items for Systematic Reviews and Meta-Analysis (PRISMA) list.^11^ To organize the process of searching for data and writing, the PICO approach was used, whose acronym represents patient (children and adolescents), intervention (social isolation), comparation (normal social coexistence), and outcome (child and adolescent development). A search was carried out in July 2020 for articles in English, Portuguese, and Spanish in the Scientific Electronic Library Online (SciELO), the Latin American and Caribbean Literature in Health Sciences (LILACS), and the PubMed databases. The descriptors used were “social isolation” AND “child development”, “quarantine” AND “child development”, “social isolation” AND “adolescent development”, “quarantine” AND “adolescent development” according to the Medical Subject Headings (MeSH), along with their equivalents for the Portuguese language, as established by the Health Sciences Descriptors (DeCS).

Original articles were included in this systematic review, without temporal delimitation, which fit as observational studies and responded to the pre-established PICO. Studies that exclusively addressed adults in their results and articles in editorial, commentary or review format were excluded. As a complementary search strategy, the bibliographic references of the articles chosen for data extraction were analyzed.

The selection of studies was made by three authors, independently and blindly, meeting the inclusion and exclusion criteria. Any differences between the authors were resolved by consensus. To analyze the chosen texts, a tabulation was made with the name of the work, year and place of publication, authors, type of study and its duration, age group of the sample, results, and limitations.

The evaluation of the methodological quality of the studies analyzed in this review was carried out based on the instrument provided by the Agency for Healthcare Research and Quality (AHRQ), which can be used in cross-sectional studies^12^ (Table 1). This checklist is composed of 11 items that also score the value of a point when completed, so the total score of an article evaluated can vary between zero and 11. Thus, the evaluation of each study was defined as: low quality (score 0–3), moderate quality (score 4–7), and high quality (score 8–11).

**Table 1:** General information about the reviewed articles.

Author/year	Location	Study design	Sample and study duration	Score*
Danese et al., 2009^6^	New Zealand	Prospective cohort	972 NB followed up until they turn 32 years of age.	8
Saurabh et al., 2020^3^	India	Retrospective cohort	252 children and adolescents aged 9–18 years (121 with quarantine experience and 131 with experience of social isolation only).	7
Sprang et al., 2013^10^	USA, Mexico, and Canada	Retrospective cohort	398 children and adolescents.	5
Schinka et al., 2013^13^	USA	Prospective cohort	832 NB followed up until they are 15 years of age.	9
Vanhalst et al, 2011^14^	Netherlands	Prospective cohort	428 adolescents aged 13–16 years followed up for five years.	7
Martin et al., 2001^15^	San Sebastian (Spain)	Prospective cohort	48 preschool children.	7
Koss et al., 2014^16^	USA	Prospective cohort	155 children between 15 and 36 months of age followed up for 25 months.	7
Caspi et al., 2006^17^	Dunedin, New Zealand	Prospective cohort	1,037 NB followed up until they were 26 years old.	5
Li et al., 2020^18^	Hebei, China	Prospective cohort	555 graduate students with an average age of 19.6 years followed up for two years.	7
Fox et al., 2011^19^	Bucharest, Romania	Prospective cohort	259 children under 30 months of age followed up until they were 8 years old (187 lived in foster care institutions and 72 lived with their families).	7
Andersen et al., 1990^20^	Norway	Prospective cohort	39 children aged 8 years of age followed up until they are 12 years old.	6
Lacey et al. 2014^21^	Great Britain	Prospective cohort	8,233 children aged 7 years of age followed up until they are 50 years old.	7

NB: newborns;

* score of the methodological quality of the studies: low quality=0 to 3, moderate quality=4 to 7, and high quality=8 to 11.

## RESULTS

**Figure 1:**
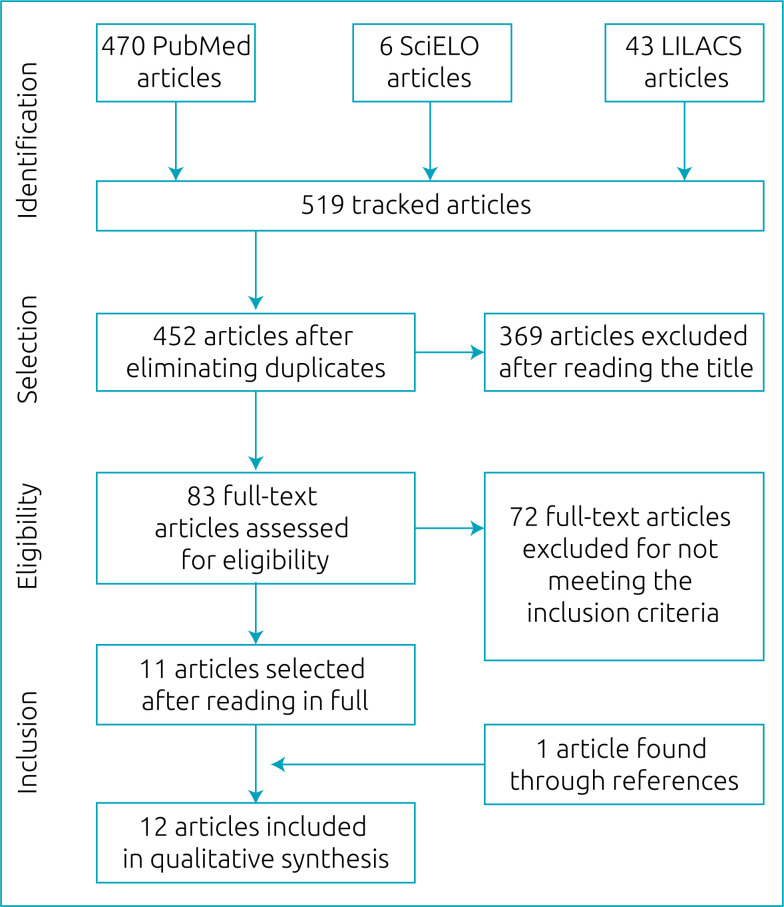
Flowchart of the review.

Initially, 519 articles were identified, and, after eliminating duplicate studies and adapting to the pre-established PICO, 436 articles were excluded. Of the 83 evaluated in full text, only 11 were included to integrate this review, as they meet all the aforementioned eligibility criteria. Subsequently, in a search through the references of the selected articles, a study met the inclusion criteria and was also selected, totaling 12 studies. The flowchart of the article selection process is shown in Figure 1.

The 12 selected articles^3^ ^,^ ^6^ ^,^ ^10^ ^,^ ^13^ ^–^ ^21^ were published between 1990 and 2020, and the studies were carried out with varied samples from countries on different continents: Oceania,^6^ ^,^ ^17^ Asia,^3^ ^,^ ^18^ Europe,^14^ ^,^ ^19^ ^–^ ^20^ and America.^10^ ^,^ ^13^ ^,^ ^16^ Only two studies were of the retrospective cohort type,^3^ ^,^ ^10^ which analyzed the impact of the pandemic caused by the coronavirus in 252 people aged 9 to 18 years old^3^ and the impacts of isolation by other pandemics in 398 children and adolescents.^10^ Among the prospective cohort articles,^6^ ^,^ ^13^ ^–^ ^21^ four^6^ ^,^ ^13^ ^,^ ^17^ ^,^ ^21^ had a duration of more than 15 years, of these, three^6^ ^,^ ^13^ ^,^ ^17^ followed the children from birth. In general, six studies worked with children and adolescents,^3^ ^,^ ^6^ ^,^ ^10^ ^,^ ^13^ ^,^ ^17^ ^,^ ^21^ four only with children,^15^ ^,^ ^16^ ^,^ ^19^ ^,^ ^20^ and two only with adolescents^14^ ^,^ ^18^ (Table 1).

To discuss this review, the authors divided the chosen studies into four categories, according to the area that the article raised as being impacted by social isolation during childhood and adolescence. Thus, the division of texts took place in five articles in the psychosocial area (Table 2),^3^ ^,^ ^10^ ^,^ ^13^ ^,^ ^14^ ^,^ ^18^ four articles in the health area in general (Table 3),^6^ ^,^ ^17^ ^,^ ^20^ ^,^ ^21^ two articles in the area of the hypothalamic pituitary adrenal axis (Table 4)^15^ ^,^ ^16^ and one article in the area of cognitive and social development (Table 4).^19^

**Table 2:** Studies on the impact of childhood isolation in the psychosocial area.

Author	Data collection/social isolation criteria	Theme	Results	Limitations
Saurabh et al.^3^	Online interview with the presence of parents. People living in a quarantine context were considered socially isolated by COVID-19.	Psychosocial	Quarantine increased the incidence of psychological problems, such as fear, nervousness, and boredom.	Sample size is small and with little socioeconomic variety among participants, mostly from immigrant families.
Sprang et al.^10^	Interview and filling of forms and checklist of PTSD by parents/guardians. People who have already lived through quarantine situations due to pandemics have entered the isolation criterion.	Psychosocial	The experience of social isolation or quarantine in childhood increased the likelihood of PTSD symptoms in this age group.	Results of the study depend on the memory of the parents who are reporting the events of years ago.
Schinka et al.^13^	Four interviews at 7, 9, 11, and 15 years of age to apply some questions and checklist of depression and social skills. Loneliness classification by the Loneliness and Social Dissatisfaction questionnaire.^36^	Psychosocial	Children with a high level of loneliness are more likely to be depressed, aggressive, and have suicidal ideation.	Very long time spans between the four questionnaires, and the result obtained was at risk of bias as it was answered by the interviewee himself.
Vanhalst et al.^14^	Five annual questionnaires, answered by the family, with a checklist application for loneliness, depressive symptoms, and personality traits. Loneliness classification by the Loneliness and Aloneness Scale for Children and Adolescents questionnaire.^37^	Psychosocial	In all stages of childhood and adolescence analyzed, there was a correlation between loneliness and the manifestation of depressive symptoms.	Low diversity among the analyzed group (mostly white and raised with the presence of both parents).
Li et al.^18^	Assessment of children and adolescents before the COVID-19 pandemic and after two weeks of isolation, using the questions from the Positive and Negative Affect Schedule (PANAS) and the Patient Health Questionnaire (PHQ-4).	Psychosocial	After two weeks of isolation, participants showed an increase in symptoms of anxiety and depression.	Low diversity among the analyzed group (mostly female undergraduate students).

PTSD: post-traumatic stress disorder.

**Table 3:** Studies on the impact of childhood isolation on health in general.

Autor	Data collection/social isolation criteria	Theme	Results	Limitations
Danese et al.^6^	Interviews with the group at 3, 5, 7, 9, 11, 13, 15, 18, 21, 26, and 32 years old and physical, psychiatric, and laboratory tests (CRP, BMI, glycated hemoglobin) at the end of the study. Evaluation of social isolation through the application of the RutterChild Scale questionnaire.^38^	Overall health	Adverse conditions experienced in childhood, such as social isolation, increase the likelihood of depression, cardiovascular disease, and metabolic diseases in adulthood.	It does not explore all the adverse conditions that a child may experience, taking into account only three of them: social isolation, precarious socioeconomic conditions, and mistreatment.
Andersen et al.^20^	Annual assessment of body size and composition, respiratory characteristics, and capacity for physical performance. Children from the periphery were considered in isolation, while those from the center had normal social life.	Overall health	The reduction in habitual physical activity caused by social isolation increased the amount of fat in the body composition and reduced the achievement of physical performance during growth.	Some of the differences that appear between children are not statistically significant. In addition, data were lacking regarding the relevance of the study.
Caspi et al.^16^	Follow-up physical, laboratory, and psychological tests were performed at the ages of 5, 7, 9, 11, 13, 15, 18, 21, and 26 years. Assessment of social isolation by applying the RutterChild Scale questionnaire.^37^	Overall health	Socially isolated children were at significantly higher risk of health problems in adulthood compared to non-isolated children.	It is not known whether the findings of this study can be generalized to all ethnic groups, as it only looks at the population of New Zealand.
Lacey et al.^20^	Follow-up of participants at intervals of 4–10 years with physical examinations, laboratory tests (CRP) and checklist of social isolation and economic factors. Assessment of social isolation by applying the RutterChild Scale questionnaire.^37^	Overall health	Socially isolated children had higher levels of CRP in middle age and were more likely to have psychological changes in adulthood.	CRP was measured only at age 44, so it is not known to what extent isolation affected CRP at each stage of development.

CRP: C-reactive protein; BMI: body mass index.

**Table 4:** Studies on the impact of isolation on childhood in the area of cognitive and social development and in the area of the hypothalamic-pituitary-adrenal axis.

Author	Data collection/social isolation criteria	Theme	Results	Limitations
Fox et al.^19^	Collection of data on children's birth and previous health. Evaluation of the relationship with the caregiver, language development and IQ at 30, 42, 54 and 96 months of age. Children in foster care institutions were considered to be in social isolation.	Cognitive and social development	Institutionalization during childhood results in the child's social isolation and becomes detrimental to his cognitive and social development.	Many of the children in the intervention and control groups were no longer residing in their original location.
Martin et al.^15^	Video recordings of behavior in interactions and play in the school environment. Evaluation of cortisol and sIgA levels in saliva samples. The criterion for isolation was the behavioral patterns presented by each child.	Hypothalamic-pituitary-adrenal axis	Social isolation was the only behavioral pattern that increased cortisol levels.	The samples were collected in only one type of situation experienced by the children (games).
Koss et al.^16^	Home collection of salivary cortisol in three samples (morning, noon, bedtime), for three days. In the same period, care in the pre-adoption period, the child's growth and socio-emotional capacity were evaluated. Children in foster care institutions were considered to be in social isolation.	Hypothalamic-pituitary-adrenal axis	Children in isolation exhibited less pronounced daytime cortisol compared to non-isolated peers of the same age; these differences did not diminish over the two-year period. It was found that early social deprivation can contribute to the early programming of the hypothalamic-pituitary axis.	Some families refused to participate in the study. Of those who agreed, not all were able to start shortly after adoption and therefore were not included in this analysis. The collection of all samples was a challenge for some of them and required several weeks, extending the time period for each collection.

IQ: intelligence quotient; sIgA: secreted immunoglobulin A.

## DISCUSSION

### Psychosocial area

Depression, as a consequence of the process of social isolation, was addressed in three studies.^13^ ^,^ ^14^ ^,^ ^18^ Vanhalst et al.^14^ established a relationship between loneliness and depressive symptoms by applying the six-item questionnaire at five different times in the development of the adolescents studied to assess the depression described by Kandel and Davies in 1982^22^ and found in all measures a correlation between the variables (Cronbach's alpha 0.34–0.49; p<0.001). In addition, Vanhalst et al.^14^ pointed out a vicious circle relationship between such factors, with loneliness being a strong predictor of depression and vice versa [chi-square(39)=170.79; p<0.001; comparative fit index (CFI)=0.92; root mean square error of approximation ([RMSEA]=0.09).

Like the study mentioned earlier, Schinka et al.^13^ also collected data on the relationship between isolation and depression. According to the study, the increasing level, when compared to the low level, of loneliness (Table 2) at 7 years of age represents a greater chance of depressive symptoms (odds ratio [OR] 1.32; p=0.004), while in adolescence, at 15 years of age, this probability becomes even greater (OR 2.05; p<0.001). The authors also collected statistical data on the association between social isolation and suicidal ideation, in the assessment made at age 15, and concluded that this relationship proved to be very strong on two occasions: in the comparison between the low level and the growing level of loneliness (OR 10.90; p=0.001), and between the low and the chronic level of loneliness (OR 18.89; p=0.009).

Still in the psychosocial area, three studies reflected on the impact that pandemics and their control measures, such as isolation and quarantine, can cause on children and adolescents.^3^ ^,^ ^10^ ^,^ ^18^ Psychological problems, including anxiety, sadness, depression, and guilt, were raised as direct consequences of the confinement process.^3^ ^,^ ^18^ Saurabh et al.^3^ pointed out how the experience of a quarantine at this age increases the incidence of worry (68.59%; p=0.069), helplessness (66.11%; p=0.039), fear (61.98%; p=0.001), and nervousness (60.33%; p=0.001) among such group. In the same line of reasoning, Li et al.^18^ evaluated the relationship between social isolation and psychological problems when comparing the average result of a checklist for positive and negative feelings (Positive and Negative Affect Schedule – PANAS),^23^ filled by adolescents before and after two weeks of confinement by COVID-19, concluding that the incidence of psychological problems was higher during the isolation experience (Cronbach's alpha 0.567; p <0.050).

Regarding the impact of pandemics, Sprang et al.^10^ evaluated the incidence of post-traumatic stress disorder (PTSD) in children and adolescents who were isolated or quarantined. The study used two checklists developed for the diagnosis of PTSD^24^ ^,^ ^25^ and showed that the isolated group is 30% more likely to enter the PTSD criteria, in relation to those who did not have such experience, with a four times bigger average score on the test among those affected. Furthermore, Sprang et al.^10^ demonstrated the fundamental role of parents in their children's behavioral development: 85.7% of parents with PTSD have children with the same symptoms, while only 14.3% of parents without PTSD have children with such disorder.

### Overall health area

Three studies bring data on how social isolation during childhood and adolescence can affect health in adulthood.^6^ ^,^ ^17^ ^,^ ^24^ The results obtained by Danese et al.^6^ showed that children with a very high level of social isolation (Table 3) were more likely to become depressed adults (relative risk [RR] 1.76; 95%CI 1.12–2.77), with a high risk of cardiac inflammation, with C-reactive protein (CRP)>3mg/L (RR 1.60; 95%CI 1.04–2.47) and with metabolic diseases such as obesity, hypertension, diabetes or hypercholesterolemia (RR 1.96; 95%CI 1.21–3.17), when compared with the reference adopted for children with a very low level of isolation (RR 1). In addition, Danese et al.^6^ pointed to social isolation as having a greater impact on the onset of diseases in adulthood than lifestyle habits such as food (RR 0.99; 95%CI 0.9–1.08), physical activity (RR 0.94; 95%CI 0.86–1.02), and smoking (RR 1.05; 95%CI 0.96–1.16).

In the same way as the study by Danese et al.,^6^ Caspi et al.^17^ described that socially isolated children had a significant risk of health problems typical of adults, such as obesity, hypertension, hypercholesterolemia, and diabetes, when compared to non-isolated children (RR 1.37; 95%CI 1.17–1.61). In addition to these, the study by Lacey et al.^21^ showed that chronic social isolation, in multiple periods of development (in childhood, adolescence, and adulthood), has a cumulative relationship in the incidence of high body mass index (BMI), smoking, and alcoholism in adults (RR 2.58; 95%CI 1.46–4.56).

Still in the area of health in general, Andersen et al.^20^ addressed the impact of isolation on health during early childhood. It was described that the children's physical activity habit depends on social contact with friends. Social isolation can generate a certain sedentary lifestyle, and children in such a condition can be harmed in their growth and functional development. In this context, isolated and non-isolated children were compared, and it was noted that the time spent on sports and leisure activities was greater in the second group. It was also found that isolated girls had a higher BMI, while boys from normal social relationships had higher estimated lean body mass. Additionally, it is worth mentioning that the maximum oxygen uptake of non-isolated children stood out, consequently, their capacity for physical performance was also better.

### Hypothalamic-pituitary-adrenal axis area

In the last few decades, there has been a significant increase in research indicating the existence of links between the immune system, the central nervous system, and the endocrine system on the one hand and psychological phenomena, such as reactions to stressful events, on the other. Some studies have attempted to investigate the relationship between social behavior in children and levels of cortisol, the main hormone produced in response to psychosocial stress, by examining the hypothalamic-pituitary-adrenal (HPA) axis. In this sense, Koss et al.^16^ examined changes in the levels of daytime cortisol in children with normal social life and isolated children and noted that the first group exhibited less pronounced rates of the hormone. In addition, children in isolation with better social care by adults and institutions had significantly higher morning cortisol (p<0.001) and more pronounced reductions in cortisol throughout the day (p<0.010), noting that social deprivation in childhood can contribute to the early programming of the HPA axis.

In the same line of research, Martin et al.^15^ analyzed the relationship between social behavior in children, their cortisol levels and their immune activity (secreted immunoglobulin A levels – sIgA). The authors researched some categories of behavior, such as isolation, solitary activity, parallel activity, proximity without interaction, social interactions, play and relationships with adults, and concluded that only isolation showed variation according to the level of cortisol (p=0.040). The group previously classified as high level for the hormone obtained the highest score for such behavior, followed by the groups of low and medium levels. Still in the same research, Martin et al.^15^ evaluated the children's sIgA levels and concluded that the immune activity was not related to the behavioral pattern in the analyzed group.

### Cognitive and social development area

Fox et al.^19^ showed that socially isolated children exhibited lower scores of intelligence quotient (IQ), compared to non-isolated children of the same age. The author states that individuals with normal social interaction had higher scores on the verbal comprehension subscale of the Wechsler Intelligence Scale for Children (WISC) (which assesses verbal and perceptual reasoning, working memory, and processing speed) at 8 years of age, compared to those children in isolation. The comparison of the results obtained by each group in the aforementioned tests was statistically significant [F(8,110)=3.09; *p*=0.004, η^2^=0.180]. In addition, it was noted that children who remained isolated until the end of the study had a significantly lower score on these scales, compared to those who returned to social life.

For a complete analysis of this review, some limitations present in the meta-analysis should be considered, such as the inclusion of an article^3^ carried out during the COVID-19 pandemic that analyzed its impacts on the development of adolescents, so it was impossible to have a complete view of the consequences in long-term isolation of young people. In addition, the present article included studies whose methodological quality was assessed with lower scores. Such an assessment is available in Table 1 for a better analysis of the data.

Despite these limitations, this systematic review brought important analyses on the impact of social isolation on the development of children and adolescents, covering the cognitive, corporal, and mental aspects in the medium and long terms. A strong relationship between such behavior and depressive symptoms has been raised, which becomes worrying as this disease, which is increasingly common among young people, is directly associated with reduced school performance,^25^ with a higher risk of smoking and suicidal ideation,^27^ and the experience of such pathology at the beginning of development makes it more likely to recur during adulthood.^28^ In addition to depression, the increase in the appearance of other psychological problems such as suicidal ideation and anxiety were raised, which reflects greater suffering and prejudice to activities related to the lives of young people. Anxiety disorders can affect school dropout, excessive use of pediatric services, given the somatic complaints related to anxiety, and the appearance of psychiatric disorders in adulthood, making social and family life more difficult.^29^ Meanwhile, suicidal ideation, which is already a growing problem in our reality, showed, in Brazil, a growth of 40% in the age group between 10 and 14 years of age and 33.5% between 15 and 19 years old in the last 10 years,^30^ becoming more alarming in the context of the pandemic.

With regard to cognitive development, it has been proven that situations of isolation during childhood impair the learning of new skills such as speaking, writing, and reading, especially in younger children,^19^ which affects school performance and makes the socialization process more difficult with colleagues, creating a vicious circle between isolation and difficulty in the learning process.^31^

In addition, isolated young people were classified as more likely to have high levels of cortisol, which is widely recognized as an impacting factor for another consequence of social isolation, the increase in BMI. The problem of changes in body composition can be easily understood when considering that children, in general, exercise in the presence and under the influence of other colleagues. Therefore, solitary behaviors lead to a sedentary lifestyle and its consequences. As for the elevated level of cortisol, it is important to highlight that it is associated with a worse performance in tests of memory and visual perception, having been observed microstructural changes in multiple areas of the brain, mainly in the splenium of the corpus callosum and in the posterior radiata corona, data the high levels of this hormone.^32^ That is, once again, social seclusion can be seen as detrimental to cognitive development in childhood and adolescence.

Some consequences that long-term isolation can have on adult health were also raised, with problems such as depression, heart disease, obesity, hypertension, diabetes, among others being highlighted. These problems already have a high incidence in adults and they often manifest together. Psychological factors such as depression and anxiety play an important role in the development and progression of heart disease,^33^ obesity,^34^ and hypertension.^35^ In addition, it should be noted that obesity is associated with an increased risk of several chronic diseases, including high blood pressure, type II diabetes mellitus, hypercholesterolemia, coronary heart disease, and others.^34^ Thus, it is evident that these factors can be intensified in adult individuals who were socially isolated during childhood.

In short, the period that corresponds to childhood and adolescence is essential for the development of the individual's physical and mental aspects. Therefore, during the pandemic, the growth of children in different areas, such as cognitive, physical, and mental, is extremely worrying. In this context, psychological assistance and the monitoring of the physical and mental health of children and adolescents is essential to reduce the damage that may be caused by social isolation.
